# Fecal microbiota in client-owned obese dogs changes after weight loss with a high-fiber-high-protein diet

**DOI:** 10.7717/peerj.9706

**Published:** 2020-10-05

**Authors:** Sandra Bermudez Sanchez, Rachel Pilla, Benjamin Sarawichitr, Alessandro Gramenzi, Fulvio Marsilio, Joerg M. Steiner, Jonathan A. Lidbury, Georgiana R.T. Woods, Alexander J. German, Jan S. Suchodolski

**Affiliations:** 1 Faculty of Veterinary Medicine, University of Teramo, Teramo, Italy; 2 Gastrointestinal Laboratory, Texas A&M University, College Station, TX, United States of America; 3 Institute of Ageing and Chronic Disease, University of Liverpool, Leahurst Neston, United Kingdom; 4 School of Veterinary Science, University of Liverpool, Leahurst Neston, United Kingdom

**Keywords:** Fecal microbiota, Canine obesity, Weight loss, 16S rRNA, Dysbiosis

## Abstract

**Background:**

The fecal microbiota from obese individuals can induce obesity in animal models. In addition, studies in humans, animal models and dogs have revealed that the fecal microbiota of subjects with obesity is different from that of lean subjects and changes after weight loss. However, the impact of weight loss on the fecal microbiota in dogs with obesity has not been fully characterized.

**Methods:**

In this study, we used 16S rRNA gene sequencing to investigate the differences in the fecal microbiota of 20 pet dogs with obesity that underwent a weight loss program. The endpoint of the weight loss program was individually tailored to the ideal body weight of each dog. In addition, we evaluated the qPCR based Dysbiosis Index before and after weight loss.

**Results:**

After weight loss, the fecal microbiota structure of dogs with obesity changed significantly (_weighted_ANOSIM; *p* = 0.016, *R* = 0.073), showing an increase in bacterial richness (*p* = 0.007), evenness (*p* = 0.007) and the number of bacterial species (*p* = 0.007). The fecal microbiota composition of obese dogs after weight loss was characterized by a decrease in Firmicutes (92.3% to 78.2%, *q* = 0.001), and increase in Bacteroidetes (1.4% to 10.1%, *q* = 0.002) and Fusobacteria (1.6% to 6.2%, *q* = 0.040). The qPCR results revealed an overall decrease in the Dysbiosis Index, driven mostly due to a significant decrease in *E. coli* (*p* = 0.030), and increase in *Fusobacterium* spp. (*p* = 0.017).

**Conclusion:**

The changes observed in the fecal microbiota of dogs with obesity after weight loss with a weight loss diet rich in fiber and protein were in agreement with previous studies in humans, that reported an increase of bacterial biodiversity and a decrease of the ratio Firmicutes/Bacteroidetes.

## Introduction

Canine obesity is a serious metabolic disease that affects the quality of life and decreases life span (Salt et al., 2019; German et al., 2012). The prevalence of obesity has been increasing in the past years in small animals (Courcier et al., 2010), and it is a major healthcare problem in veterinary practice (Chandler et al., 2017; German, 2006). Obesity is associated with a greater risk of developing other diseases such as diabetes mellitus, cardiovascular and orthopedic diseases, and even some types of cancer (Kopelman, 2000; Tropf et al., 2017; German et al., 2010b). Diet restriction increased life span in dogs and weight loss regimen based on weight loss diet and exercise decreased plasma insulin concentrations and insulin:glucose ratio (Kealy et al., 2002; German et al., 2009). Due to the detrimental effect of obesity on the welfare of both dogs and their owners, investigating new approaches to prevent obesity and promote weight loss in small animals is of crucial interest in veterinary research (Day, 2017; Bartges et al., 2017).

In the past years, there has been interest on investigating a possible role for gut microbiota in obesity in humans, mouse models (Zhao, 2013; Turnbaugh et al., 2008; Ridaura et al., 2013), and also in dogs (Forster et al., 2018; Handl et al., 2013; Park et al., 2015; Kieler et al., 2017; Salas-Mani et al., 2018). Studies have found that obesity is associated with alterations, disruption, and decreased biodiversity of the intestinal microbiota (Durack & Lynch, 2019; Ley et al., 2005; Ley et al., 2006; Cotillard et al., 2013). In addition, colonization of germ-free mice with the fecal microbiota of obese humans lead to significant weight gain when compared to mice that received fecal microbiota from lean controls (Ridaura et al., 2013), suggesting that gut microbiota impacts host physiology and metabolism.

While a relationship between the gut microbiome and obesity has been observed, it remains unclear as to how the gut microbiome contributes to the development of obesity, but proposed mechanisms include the production of short chained fatty acids (SCFAs), monosaccharides, and other bioactive molecules. These bacterial products may lead to an increase in dietary energy harvest (Turnbaugh et al., 2006), changes in lipid metabolism (Ghazalpour et al., 2016), changes in fat storage regulation (Bäckhed et al., 2004; Bäckhed et al., 2007), altered satiety (Arora, Sharma & Frost, 2011), and an increase in systemic low-grade inflammation via the interaction with either the enteric nervous system (Schwartz, 2000; Tehrani et al., 2012; De Lartigue, De La Serre & Raybould, 2011), the endocrine system (Mondo et al., 2020; Kirchoff, Udell & Sharpton, 2019; Scarsella et al., 2020), or the immune system (Cani et al., 2007; Cani et al., 2012).

In human and animal models of obesity, a greater abundance of the phylum Firmicutes and lesser abundance of Bacteroidetes have been reported (Turnbaugh et al., 2009; Vrieze et al., 2012), and the Firmicutes/Bacteroidetes (F:B) ratio is commonly used as a marker of gut microbial dysbiosis in obesity. The F:B ratio is greater in individuals with obesity and, interestingly, decreases after weight loss (Ley et al., 2005; Ley et al., 2006). Previous data have shown similarities in the gut microbiota of humans and dogs (Swanson et al., 2011; Coelho et al., 2018), but it is not clear whether results from human and animal models can be translated to canine obesity.

One study in research Beagles evaluated the fecal microbiota of lean dogs and dogs that developed obesity after overfeeding for 6 months. Analysis of fecal microbiota demonstrated differences in microbial communities between dogs in the obese and lean groups, with a lesser diversity in the obese group. In particular, there was a lesser abundance of Firmicutes and Fusobacteria in the obese group, and the abundance of Proteobacteria was significantly greater in the obese group compared to the lean group (Park et al., 2015).

In one study of client-owned dogs, a dominance of the phylum Firmicutes has been seen, with significant differences at the taxonomic level, between dogs with obesity and those in ideal body condition, but no significant differences in the overall composition of fecal microbiota or bacterial diversity (Handl et al., 2013). However, in a second study, a trend towards lower fecal microbial diversity was seen in dogs with obesity, compared with dogs in ideal bodyweight (Forster et al., 2018).

Two studies have evaluated the impact of weight loss on the fecal microbiota of dogs with obesity. In one study, the fecal microbiota was assessed before, during, and after 12 weeks of a weight loss program that consisted of diet and exercise or diet alone. Despite the short follow-up period, differences in bacterial abundance were identified after 6 weeks and 12 weeks of the weight loss program. While not all the dogs lost as much weight as expected, a decrease in *Megamonas* and an unknown genus of the family Ruminococcaceae was observed in the dogs with a higher weight loss rate (Kieler et al., 2017). The fecal microbiota composition of research Beagles with obesity has also been assessed before and after a 17-week weight loss program with a hypocaloric diet (Salas-Mani et al., 2018). Despite all dogs reaching ideal body weight, no significant impact on diversity was seen and microbial communities remained similar to baseline values after 17 weeks. At the genus level, significant differences were found only in the abundances of the Firmicutes genera *Lactobacillus*, *Clostridium*, and *Dorea*, which decreased after the weight loss program, and *Allobaculum*, which increased (Salas-Mani et al., 2018).

A number of limitations need to be considered in these studies. Microbiome analysis evaluates a large number of variables, which limits the statistical power, especially in small cohorts (Falony et al., 2016). In addition, studies with healthy client-owned dogs have identified large individual variations, which need to be taken into account (Garcia-Mazcorro et al., 2012). Given that obesity develops over time, it is reasonable to expect that significant changes will be seen only when follow-up focuses on the long-term improvement. Therefore, the aim of this study was to use 16S rRNA sequencing to evaluate the differences in fecal microbiota composition of client-owned dogs with obesity before and after weight loss. We also performed quantitative PCR to calculate the Dysbiosis Index in obese dogs before and after weight loss and to compare the values obtained with the established reference intervals from healthy dogs (AlShawaqfeh et al., 2017). Moreover, we evaluated the fecal microbiota of obese client-owned dogs enrolled in the weight loss program that did not reach ideal body weight to quantify the effect of the diet alone on the fecal microbiota.

## Materials & Methods

### Study Animals, eligibility criteria and ethical considerations

Client-owned dogs with obesity were referred to the Royal Canin Weight Management Clinic, University of Liverpool, UK. All were presented between June 2009 and August 2017, and completed their weight loss regimens between November 2009 and August 2018. To be included in the study, dogs had to be clinically healthy with no signs of gastrointestinal disease, a BCS of ≥6, and no antimicrobial usage in the past month (Igarashi et al., 2014). None of the dogs had antimicrobials throughout their weight loss program. No fecal analyses were performed on the fecal samples and so occult infection with intestinal parasites could not be excluded. However, no dog showed signs consisted with parasitic infection.

Historical data and fecal samples, before and after participation in the weight loss program, had to be available for the analysis. The final number of dogs with obesity that met the inclusion criteria was 25. At time point zero (T0), fecal samples of all 25 dogs were collected. Twenty dogs completed the weight loss program and reached their target weight, whilst five dogs stopped their program early as request of the owners for undeclared reasons. From dogs that completed the weight loss, a fecal sample from the visit in which they reached their target body weight, was collected to include in the analysis as time point two (T2). Dogs that did not complete the weight loss program but had a fecal sample from the first follow-up visit (time point one (T1)) were included in the analysis to account for the effect of the new diet on the fecal microbiota. The study protocol was reviewed and approved by the University of Liverpool Veterinary Research Ethics Committee (Approval reference: RETH000353 and VREC793), the Royal Canin ethical review committee, and the WALTHAM ethical review committee. Owners of dogs with obesity gave informed consent in writing.

### Weight loss regimen

Prior to commencing weight reduction, all dogs were considered to be healthy apart from their obesity. All dogs were screened for overall health by performing complete blood count, serum biochemical analysis, serum free thyroxine concentration (by equilibrium dialysis) and urinalysis. Dogs were weighed at admission, and the body condition score (BCS) was estimated using a 9-integer scale by the attending clinician (AJG). Percentage body fat was measured by dual-energy X-ray absorptiometry (DEXA) as previously described (Raffan et al., 2006). For weight reduction, all dogs were fed a dry therapeutic diet (Canine Satiety^®^ diet, Royal Canin), with the exception of one dog (OBE16) that was fed a combination of wet and dry food (Canine Satiety^®^ diet, Royal Canin). Moreover, the formulation of Satiety changed in 2010 (Table 1, Data S1). The endpoint of the study was achievement of ideal body weight which, given differing degrees of adiposity, was individually set for each dog using the results of body composition analysis from before weight loss, as previously reported (German et al., 2012). Briefly, pre-weight-loss body composition data were entered into a computer spreadsheet which contained a bespoke mathematical formula to predict ideal bodyweight. The formula was based upon typical changes in body composition seen from previous weight loss studies at the same clinic (German et al., 2007; German et al., 2010a).

**Table 1:** Average composition of diets for weight loss. Diet formulation changed in 2010; figures in column refer to pre-2010 and post-2010 diets, respectively.

Criterion	Dry food^a^		Wet food^b^	
ME content	2,900/2,865 Kcal/kg		602 Kcal/kg
	Per 100g AF	g/1,000 Kcal (ME)	Per 100g AF	g/1,000 Kcal (ME)
Moisture	8/10	28/33	83	1,379
Crude protein	30/30	103/105	8.5	141
Crude fat	10/10	33/33	2.0	33
Starch	19/18	66/61	1.8	30
NFE	30/29	102/100	3.0	50
Crude fiber	18/16	60/58	2.0	33
Total dietary fiber	28/28	97/97	3.2	53
Ash	5.3/5.7	18/20	1.5	25

**Notes.**

ME Metabolizable energy content, as measured by animal trials according to the American Association of Feed Control Officials protocol (AAFCO, 2010) AF as fed DM dry matter

a Satiety Support Canine Dry (Royal Canin).

b Satiety Support Canine Wet (Royal Canin).

### Fecal collection and DNA extraction

Fecal samples from dogs with obesity were collected after spontaneous defecation and stored at −20 °C being shipped to the Gastrointestinal Laboratory at Texas A&M University in February 2019. DNA was extracted from approximately 100 mg of stool using the Mo Bio PowerSoil^®^ DNA isolation kit (MoBio Laboratories, USA) according to the manufacturer’s instructions.

### Quantitative PCR (qPCR) and Dysbiosis Index (DI)

Quantitative PCR was performed using universal bacteria primers and primers for the following bacterial groups: *Blautia* spp., *Clostridium hiranonis (C. hiranonis)*, *Escherichia coli (E. coli)*, *Faecalibacterium* spp., *Fusobacterium* spp., *Streptococcus* spp., and *Turicibacter* spp. The analysis was performed using a CFX 96 Touch TM Real-Time PCR Detection system (Biorad Laboratories). Ten µL SYBR-based reaction mixtures: 5 µL of SsoFast™ EvaGreen^®^ Supermix (Biorad Laboratories), 2.2 µL of water, 0.4 µL of each primer (final concentration: 400 nM), and 2 µL of DNA (1: 10 or 1: 100 dilution) were used for a protocol of 95 °C for 2 min, and 40 cycles at 95 °C 5 s and 10 s at the optimized annealing temperature for each primer set. Afterwards, a melt curve analysis was completed (AlShawaqfeh et al., 2017).

Results from the qPCR analysis for *Blautia* spp., *C. hiranonis*, *E. coli*, *Faecalibacterium* spp., *Fusobacterium* spp., *Streptococcus* spp., and *Turicibacter* spp. are expressed as the abundance of DNA for each bacterial group (logarithm of starting quantity or logarithm of relative DNA copy number). Relative DNA copy number for the mentioned bacteria were used to calculate a single numerical value known as the Dysbiosis Index (AlShawaqfeh et al., 2017). A value < 0 is indicative of a normal microbiota, numbers between 0 and 2 are considered equivocal, while numbers greater than 2 indicate fecal dysbiosis. The dysbiosis index is a commercially available assay, and the reference intervals have been validated with dogs from various countries, including the UK.

### 16S rRNA gene sequencing

The V4 variable region of the 16S rRNA gene was sequenced at the MR DNA laboratory (http://www.mrdnalab.com, Shallowater, TX, USA). Primers 515F (5′-GTGYCAGCMGCCGCGGTAA) (Parada, Needham & Fuhrman, 2016) to 806RB (5′-GGACTACNVGGGTWTCTAAT) (Apprill et al., 2015) and HotStarTaq Plus Master Mix (Qiagen, USA) were used to amplify samples and perform the Illumina MiSeq protocol following the manufacturer’s guidelines. Raw sequences were uploaded into Sequence Read Archive of the NCBI GenBank database under the accession number PRJNA580258.

### Analysis of sequences

Quantitative Insights into Microbial Ecology 2 (QIIME 2.0) was used for analysis of the 16S rRNA amplicon sequences (Bolyen et al., 2019). Sequences were demultiplexed and the OTU table was created using DADA2 (Callahan et al., 2016). Operational taxonomic units (OTUs) were defined as sequences with at least 97% similarity within the Greengenes v 13.8 database (DeSantis et al., 2006). Prior to downstream analysis, sequences assigned as chloroplast, mitochondria, and low abundance OTUs, containing less than 0.01% of the total reads in the dataset were removed (McDonald et al., 2012). All samples were rarefied to an even depth of 36,775 sequence reads, based on the lowest read depth of samples.

Alpha diversity was evaluated with Chao1, Shannon diversity, and observed OTUs. Beta-diversity metric was estimated by unweighted and weighted phylogeny-based UniFrac distances and visualized using PCoA (Principal Coordinate Analysis) plots.

### Statistical analysis

Normality was tested for all continuous variables using the Shapiro–Wilk test. Results were reported as mean (standard deviation [SD]) or median (range), when data were normally- or not normally-distributed, respectively. Differences in dog characteristics (i.e., age) between groups at baseline were compared using either the *t*-test. The ANOSIM (Analysis of Similarity) test within PRIMER 6 software package (PRIMER-E Ltd., Luton, UK) was used to analyze significant differences in microbial communities between groups.

Alpha diversity indices (Shannon, Chao1, and Observed OTUs), Dysbiosis Index, and quantitative PCR results were compared between groups using Wilcoxon test. A statistical software package (Prism version 8.0; GraphPad Software, San Diego, CA, USA), was used for the described analyses. To minimize false discoveries in univariate statistics, OTUs not present (0%) in at least 50% of the samples of at least one of the compared groups were considered rare and excluded from analysis. Filtered taxa were tested with Wilcoxon test for paired analysis using statistical software (R Studio Software version 1.2.1335 ©, R Studio, Boston, MA, USA; and JMP Pro 14; SAS, Durham, NC, USA). *P*-values were adjusted using the Benjamini–Hochberg Step-up method with a false discovery rate (FDR) of 0.05. For all statistical analyses, significance was set at *p* < 0.05.

## Results

### Animal population characteristics

After a mean of 330.9 (SD 203.4) days on weight loss diet, BCS changed significatively in dogs with obesity when compared before (BCS 8; range 6–9) and after weight loss (median BCS 5; range 4–7; *p* < 0.001). However, BCS was not significantly different at baseline between the group of dogs that lost weight and those that did not complete the weight loss program ( *p* = 0.551). At baseline, the mean age in dogs with obesity that lost weight was 69.4 (SD 32.3) months, and 75.0 (SD 23.6) months for those that did not lose weight (*p* = 0.730), (Table 2, Data S1). The breeds included in the obese group were: Labrador Retriever (*n* = 7), Golden Retriever (*n* = 2), Cavalier King Charles spaniel (*n* = 2), Border Collie (*n* = 2), Pug (*n* = 2), Lhasa Apso (*n* = 1), American Bulldog (*n* = 1), Dachshund (*n* = 1), Rottweiler (*n* = 1), Newfoundland (*n* = 1), Bichon Frise (*n* = 1), Rough Collie (*n* = 1), and mixed breed (*n* = 3).

**Table 2:** Demographics for dogs with obesity enrolled in this study. Unpaired *T*-test was used to compare age between the groups of obese dogs that completed the study vs. obese dogs that did not complete the study (*p* = 0.730). Mann-Whitney for unpaired analysis and Wilcoxon for paired analysis were used to test significance of differences in BCS between groups: obese before vs. after weight loss (*p* < 0.001); obese dogs that completed the program at baseline vs. obese dogs that did not complete the study (*p* = 0.551).

	Days on weight loss diet, Mean (SD)	Age in months, Mean (SD)	Sex	Sexual status	BCS Baseline, median (range)	BCS after weight loss, median (range)	BCS during weight loss, median (range)
Obese dogs, completed study (*n* = 20)	330.9 (SD 203.4)	69.4 (SD 32.3)	10F/10 M	18N/2I	8 (6–9)	5 (4–7)	N/A
Obese dogs, did not complete study (*n* = 5)	536.4 (SD 154.8)	75 (SD 23.6)	3F/2M	5N	8 (8–9)	N/A	8 (8–9)

**Notes.**

F female M male N neutered I intact BCS Body condition score

Body composition measurements were available for 19 out of the 20 dogs with obesity that lost weight (*n* = 20) and mean body fat mass was 44.8% (SD 5.0%) before weight loss (T0) and 30.4% (SD 6.5%) after weight loss (T2, *p* < 0.001). Mean rate of weight loss of starting body weight was 0.68% (SD 0.29%) per week, while the energy intake during the weight loss period was 60.8 (SD 5.5) kcal per kg^0.75^ of ideal body weight per day (Table 2, Data S1).

Dogs were classified as having discontinued prematurely (*n* = 5) when their body weight at the time that they dropped out of the study (T2) were still significantly above of their ideal weight. The time between enrollment (T0) and the first follow-up (T1) was 15 (14–37) days, during which they had lost a median of 2.7% (0.0%–4.8%) of their starting body weight. At the time they dropped out of the program, after 414 (414–781) days on weight loss, they lost 9.9% (−3.6%–21.3%) of body weight. Although one dog did eventually lose 21.3% of its body weight, it was still significantly (∼13%) above its ideal weight at the time it discontinued and had also not lost any weight when the follow-up fecal sample was taken. All other dogs in this group lost <11% of their starting weight and were also above their ideal weights at the point that their weight loss was ended. The median rate of weight loss of starting body weight was 0.10% (−0.06%–0.39%) per week and the energy intake was 54.0 (51.8%–60.9%) kcal per kg^0.75^ of ideal body weight per day. Body composition measurements were available in 4 out of 5 of the dogs at baseline, and median body fat was 44.7% (44.0%–47.5%). Body condition score did not change significatively during the period of weight loss (Data S1). No significant differences were identified for alpha and beta diversity when sex, age and neutered status were investigated for association with fecal microbiota in all dogs at baseline (Data not shown).

### qPCR and dysbiosis index

**Figure 1:**
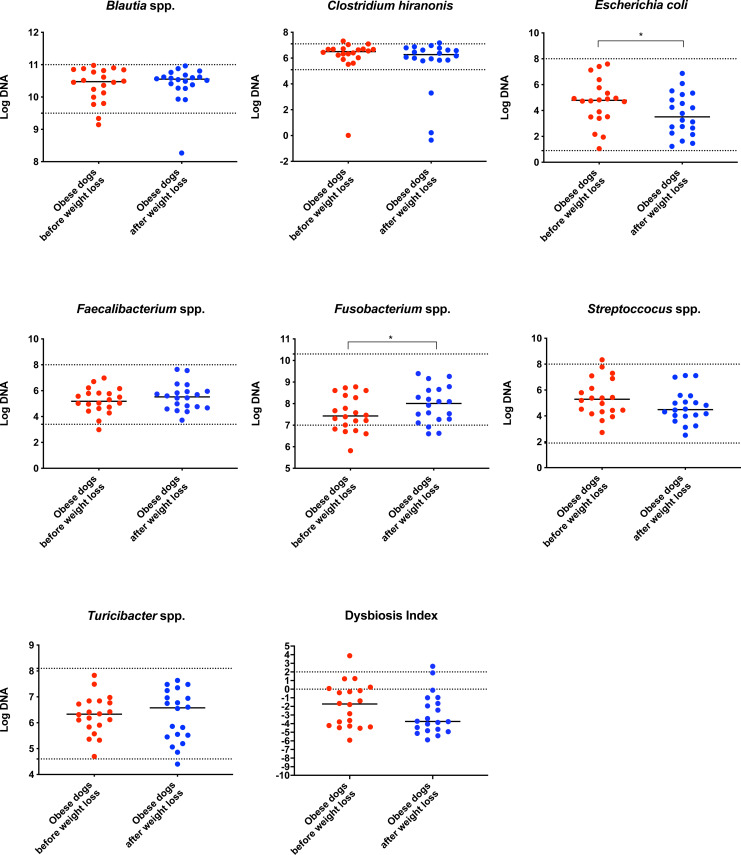
Dysbiosis Index and quantitative PCR results for *Blautia* spp., *C. hiranonis*, *E. coli*, *Faecalibacterium* spp., *Fusobacterium* spp., *Streproccocus* spp., and *Turicibacter* spp. Bacterial concentrations are expressed in Log DNA (Log of the starting quantity, which is the relative DNA copy number). The dotted lines indicate the established reference intervals for each bacterial group for clinically healthy dogs. The Dysbiosis Index is a mathematical algorithm that summarizes the resuts in one number. A negative value is indicative of a normal microbiota, numbers between 0 and 2 are considered equivocal, and values greater than 2 indicate dysbiosis. Wilcoxon test was used to compare bacterial abundance and Dysbiosis Index between dogs with obesity before and after weight loss. Significance ^∗^*p* < 0.05, ^∗∗^*p* < 0.01, ^∗∗∗^*p* < 0.001.

On analysis by qPCR, there was a decreased abundance of *E. coli* (T0: 4.8 vs. T2: 3.5; *p* = 0.030), and an increased abundance of *Fusobacterium* spp. (T0: 7.4 vs. T2: 8.0; *p* = 0.017) after weight loss (Fig. 1). The values for the abundances of the evaluated bacteria by qPCR and for the Dysbiosis Index showed that the changes observed in the fecal microbiota before and after weight loss were mostly within the established reference intervals for clinically healthy dogs.

### Changes in the fecal microbiota with weight loss in dogs with obesity

**Figure 2:**
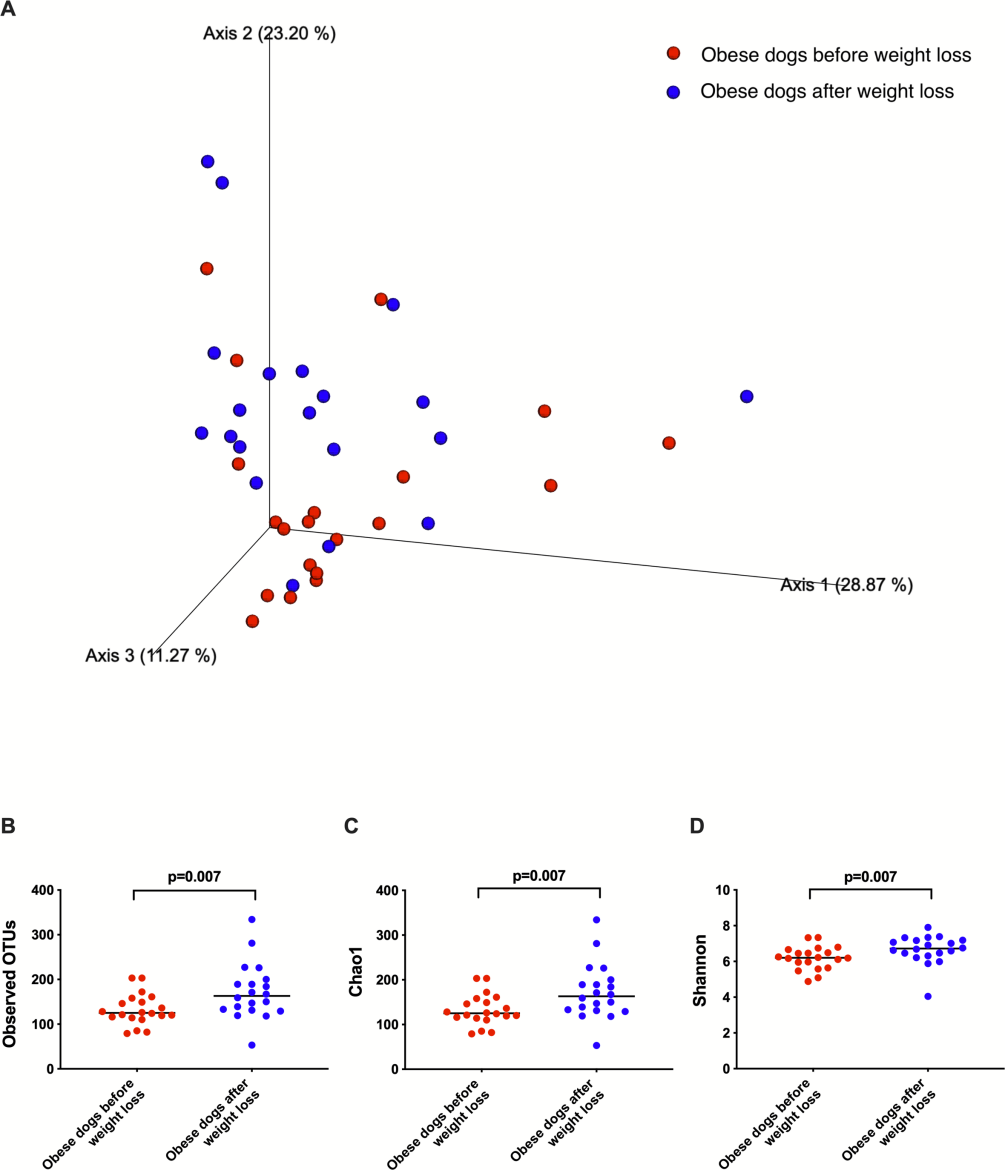
Principal coordinate analysis of beta and alpha diversity of dogs with obesity before and after weight loss. (A) PCoA plot based on weighted UniFrac distances of 16S rRNA genes. Visible clustering was confirmed by ANOSIM, showing that fecal microbiota of obese dogs changed significatively after weight loss (*p* = 0.016, *R* = 0.073). (B) Observed OTUs, an indicator of species richness, and (C) Chao1, indicator of rare bacterial species abundance showed an increase after weight loss (*p* = 0.007). (D) Shannon index, indicator of bacterial evenness, also increased significatively when dogs lost weight (*p* = 0.007).

Weighted UniFrac analysis of similarities revealed significant clustering of the microbial communities in dogs with obesity before and after weight loss (weighted ANOSIM; *p* = 0.016, *R* = 0.073; Fig. 2A). Alpha diversity evenness and richness, as indicated by Shannon (*p* = 0.007), Chao1 (*p* = 0.007), and Observed OTUs (*p* = 0.007) indices were significantly increased after weight loss (Fig. 2B).

**Figure 3:**
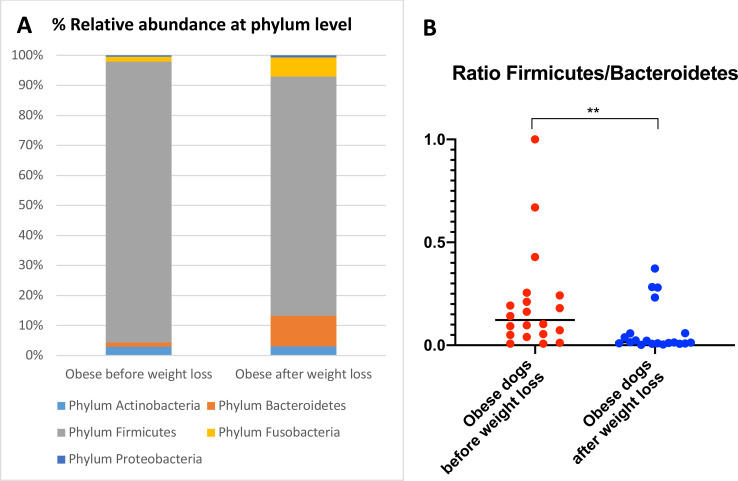
Abundance of fecal bacteria at phylum level found in obese dogs before and after weight loss. (A) Relative abundance of the phyla detected in fecal samples of obese dogs before weight loss and after weight loss. Increases of the abundance of the phyla Bacteroidetes (*q* = 0.002) and Fusobacteria (*q* = 0.040), and decreases of the abundance of the phylum Firmicutes (*q* = 0.001) were observed after weight loss. (B) Firmicutes/Bacteroidetes ratio values for each dog. After weight loss, Firmicutes/Bacteroidetes ratio decreased significantly as a consequence of a greater abundance of Bacteroidetes and lesser of Firmicutes (*p* = 0.004).

**Figure 4:**
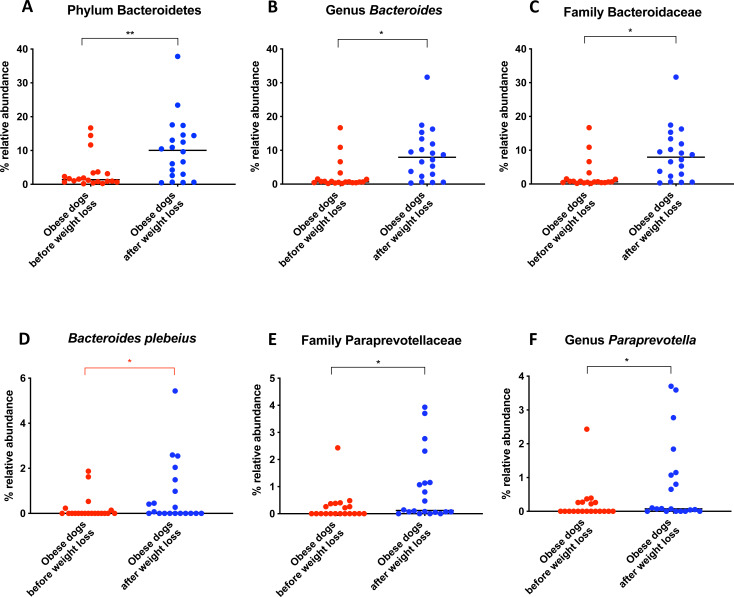
Relative abundance of bacterial populations belonging to the phylum Bacteroidetes detected in fecal samples of obese dogs that changed after weight loss. Significance ^∗^*p* < 0.05, ^∗∗^*p* < 0.01, ^∗∗∗^*p* < 0.001. Red significance lines indicate *p*-values that did not pass multiple comparison correction.

**Figure 5:**
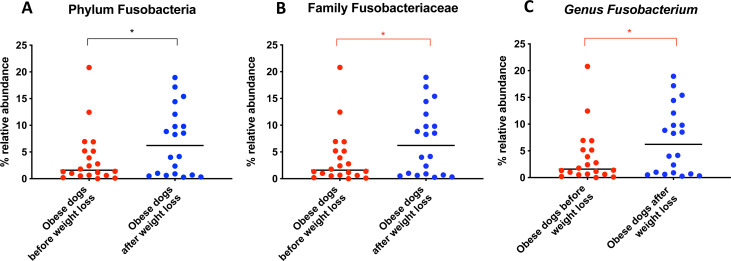
Relative abundance of bacterial populations belonging to the phylum Fusobacteria detected in fecal samples of obese dogs that changed after weight loss. Significance ^∗^*p* < 0.05, ^∗∗^*p* < 0.01, ^∗∗∗^*p* < 0.001. Red significance lines indicate *p*-values that did not pass multiple comparison correction.

**Figure 6:**
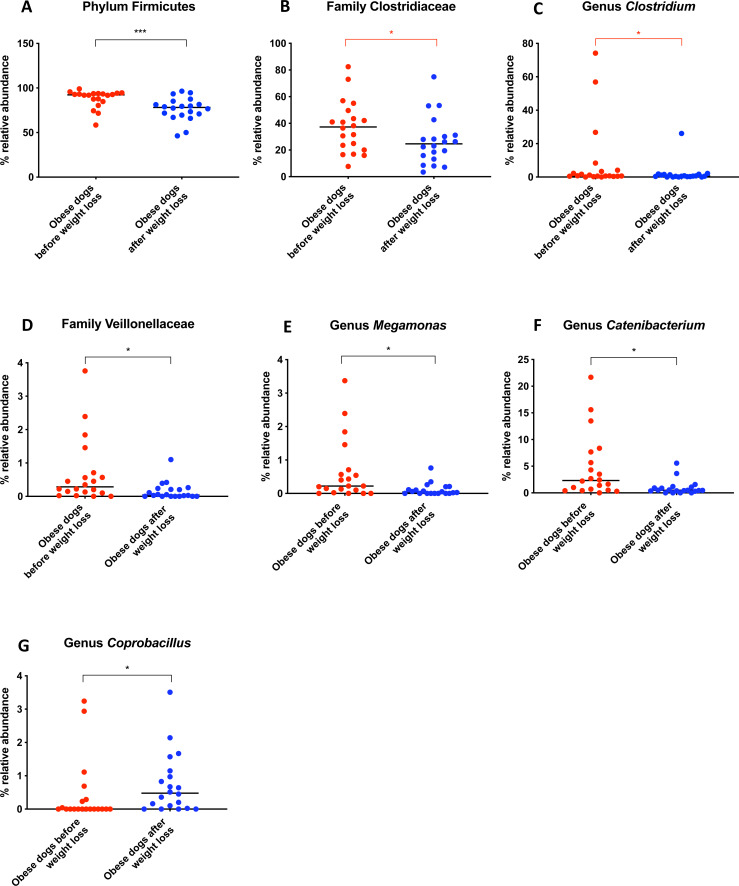
Relative abundance of bacterial populations belonging to the phylum Firmicutes detected in fecal samples of obese dogs that changed after weight loss. Significance ^∗^*p* < 0.05, ^∗∗^*p* < 0.01, ^∗∗∗^*p* < 0.001. Red significance lines indicate *p*-values that did not pass multiple comparison correction.

When the bacterial abundance was investigated at the taxa level, significant differences were found for the phyla Bacteroidetes, Firmicutes and Fusobacteria (Fig. 3A). The median Firmicutes/Bacteroidetes ratio decreased from 0.123 to 0.014 (*p* = 0.004) in dogs with obesity after weight loss (Fig. 3B). The relative abundance of Bacteroidetes (T0: 1.4% vs. T2: 10.1%; *q* = 0.002; Fig. 4A) and Fusobacteria (T0: 1.6% vs. T2: 6.2%; *q* = 0.040; Fig. 5A) increased significantly after weight loss, whilst the abundance of the phylum Firmicutes, instead decreased (T0: 92.3% vs. T2: 78.2%; *q* = 0.001; Fig. 6A).

The increase in Bacteroidetes was driven by the genera *Bacteroides* (T0: 0.7% vs. T2: 7.9%; *q* = 0.017; Fig. 4C) and *Paraprevotella* (T0: 0% vs. T2: 0.1%; *q* = 0.033; Fig. 4F).

From the phylum Fusobacteria, the genus *Fusobacterium* (T0: 1.6% vs. T2: 6.2%; *q* = 0.099; Fig. 5C) increased after weight loss. However, the corrected *q*-value did not reach significance.

Belonging to the phylum Firmicutes, the family Clostridiaceae decreased in abundance after weight loss (T0: 37.3% vs. T2: 24.6%; *q* = 0.068), but this difference did not reach significance after Benjamini correction (Fig. 6B). The same was noticed for the genus *Clostridium* (T0: 0.7% vs. T2: 0.6%; *q* = 0.119; Fig. 6C). The genus *Megamonas* (T0: 0.2% vs. T2: 0.0%; *q* = 0.027; Fig. 6E) and the genus *Catenibacterium* (T0: 2.3% vs. T2: 0.5%; *q* = 0.017) decreased significantly after weight loss (Fig. 6F), and the genus *Coprobacillus* increased in abundance (T0: 0% vs. T2: 0.5%, *q* = 0.033; Fig. 6G).

### A short-term change in diet does not alter fecal microbiota

**Figure 7:**
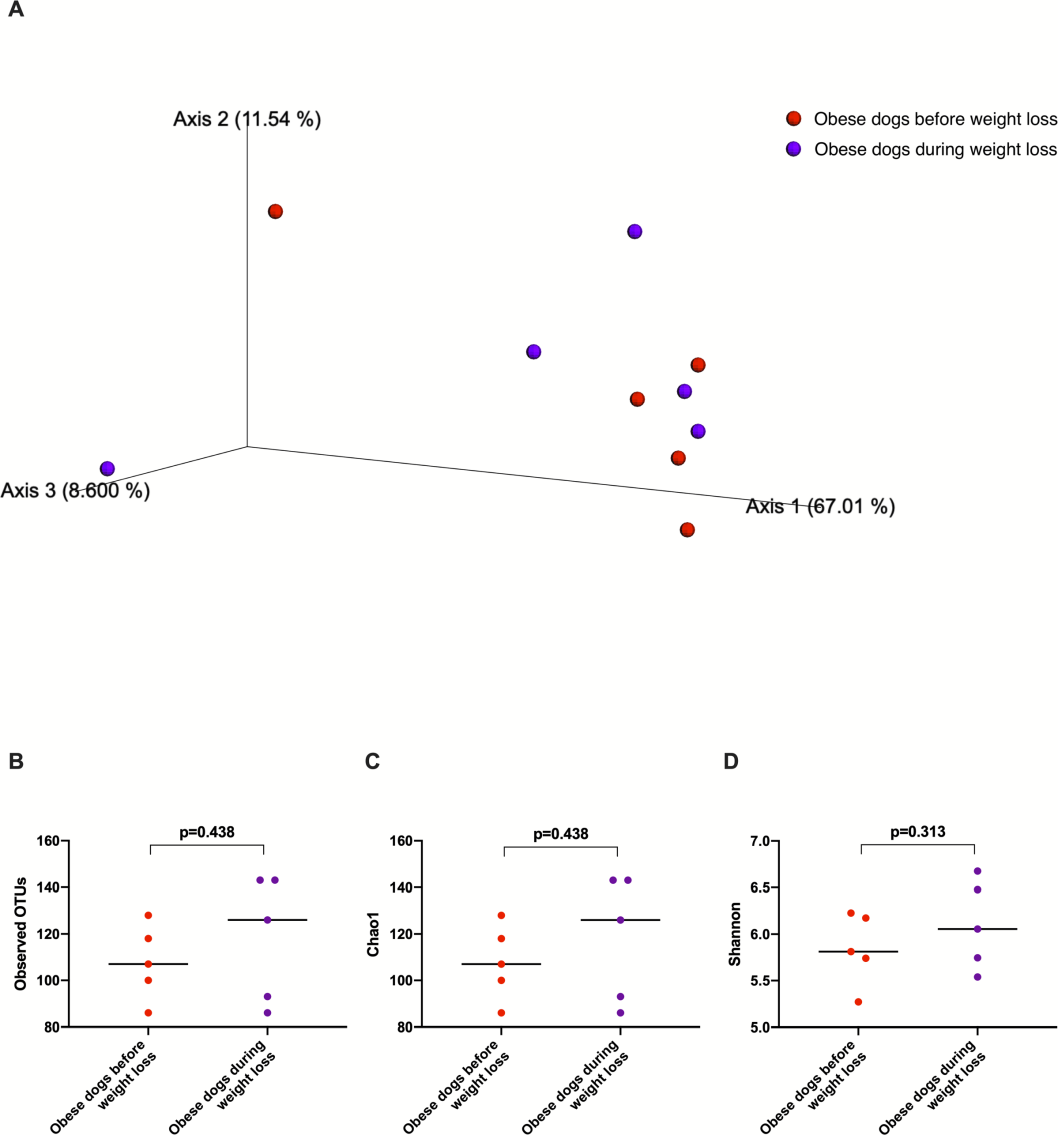
Principal coordinate analysis of beta diversity and alpha diversity indices of obese dogs that did not complete the weight loss program. (A) PCoA plot based on weighted UniFrac distances of 16S rRNA gene shows no clustering of microbial communities from feces of obese dogs before weight loss (red) and during weight loss (purple). Fecal microbiota profile of obese dogs did not change after a median period of 15 days weighted ANOSIM; *p* = 0.778, *R* =  − 0.080). (B) Observed OTUs, an indicator of species richness, (C) Chao1, indicator of rare bacterial species abundance (*p* = 0.438), and (D) Shannon index, indicator of bacterial evenness (*p* = 0.313), were not different in obese dogs after a median period of 15 days on weight loss program.

The fecal microbiota beta diversity of the dogs with obesity that stopped the weight loss program before reaching the endpoint (*n* = 5) was analyzed before and during the weight loss program. No significant differences were evident between the two time points (Weighted ANOSIM; *p* = 0.778 *R* =  − 0.080; Fig. 7A). Alpha diversity evenness and richness, as indicated by Shannon (*p* = 0.313), Chao1 (*p* = 0.438), and Observed OTUs (*p* = 0.438) indices did not show significant differences after a median period of 15 days on weight loss diet.

## Discussion

In this study, we report significant differences in the fecal microbiota in a population of 20 obese client-owned dogs after weight loss. Dogs with obesity were enrolled in a weight loss program with the endpoint set as the achievement of target body weight (German et al., 2007; German et al., 2010a; German et al., 2010b).

We observed that the fecal microbiota richness and evenness of dogs increased significantly after weight loss, which is consistent with previous studies for dogs and humans with obesity, where a lesser richness and evenness of the fecal microbiota was reported in obese individuals (Park et al., 2015; Peters et al., 2018). At the phylum level, our results showed a decrease of the abundance of Firmicutes (92.3% vs. 78.2%) and an increase of the abundance of Bacteroidetes (1.4% vs. 10.1%) after weight loss, as a result, we observed a decrease of the F:B ratio in dogs with obesity after weight loss (Fig. 3B). This is consistent with the literature (Ley et al., 2006), since the F:B ratio of obese individuals has been reported to be greater in studies that analyzed the fecal microbiota of obese humans, dogs, and animal models of obesity, that also decreased after weight loss (Ley et al., 2005; Ley et al., 2006; Turnbaugh et al., 2006; Turnbaugh et al., 2009; Salas-Mani et al., 2018).

An important difference of the core microbiota between humans and dogs is the abundance of the phylum Fusobacteria. In human studies, Fusobacteria is not as abundant in the fecal microbiota compared to dogs (Swanson et al., 2011; Coelho et al., 2018). In fact, in humans, a high abundance of Fusobacteria is associated with colon cancer (Kelly, Yang & Pei, 2018). In contrast, in dogs, Fusobacteria appears to play an important role in the maintenance of health, and has been reported to be decreased in dogs with gastrointestinal diseases (AlShawaqfeh et al., 2017; Minamoto et al., 2014). Previous studies have demonstrated that a greater abundance of Fusobacteria is associated with leanness and it increases after weight loss in dogs (Park et al., 2015; Handl et al., 2013). Our results by 16S rRNA gene sequencing confirm that the abundance of Fusobacteria increases after weight loss (Fig. 5A).

In agreement with this, results from qPCR (Fig. 1) showed also a significant increase in *Fusobacterium* spp. and a significant decrease in *E. coli*, with a numerical decrease in the Dysbiosis index, although this was not significant. Most dogs remained within the established reference interval for clinically healthy dogs.

A greater abundance of the class Clostridia has been associated with an obese phenotype and it is reported to decrease after weight loss in humans (Nadal et al., 2009). In a similar study in dogs, the genus *Clostridium* decreased after a weight loss program of 17 weeks (Salas-Mani et al., 2018). Our results also showed a slight decrease of *Clostridium* after weight loss. However, this difference was not statistically significant (Fig. 6C).

One of the factors to consider may be the variability between diets. In order to evaluate the effect of the weight loss diet in the gut microbiota, fecal microbiota of dogs with obesity before and after an initial period on the weight loss diet was analyzed in a small cohort of dogs with obesity that did not complete the weight loss program. The fecal microbiota analysis before and after this period did not show significant differences (Fig. 7). Despite the short-term on weight loss diet and the small sample size, similar results were shown in the study carried out by Kieler and colleagues (Kieler et al., 2017), that evaluated the fecal microbiota of overweight pet dogs after a weight loss program. In addition, the same weight loss diet used in our study was used with or without exercise in the mentioned study, and researchers observed only minor changes in the microbiome composition. In that particular study, the dogs were followed for 12 weeks, and it is not clear how many dogs reached an ideal body weight. The main finding was that a decrease in abundance of the genus *Megamonas* correlated with a greater weight loss rate during 12-week weight loss program (Kieler et al., 2017). We also observed a decrease in the genus *Megamonas* after weight loss, which could be attributed to an effect of the weight loss diet. However, the role of *Megamonas* in obesity is unclear and merits further investigation.

There is significant interest in body weight management by modifying macronutrient distribution in diets. Fiber promotes digestive health and weight control and has been demonstrated to improve satiety in dogs (Weber et al., 2007), however, the effect of diet on fecal microbiota in dogs is controversial. Whilst diet has been shown to modulate the gut microbiota in humans (David et al., 2014), in dogs this correlation is not always clear. Gut microbiota seems to be modulated by diet only when its formulation changes significantly in macronutrient content from the previous diet (Schmidt et al., 2018; Kim et al., 2017) or the intestinal microbiota is compromised due to a gastrointestinal disease (Bresciani et al., 2018). Consistently, in healthy dogs, small variations in diets seem not to have substantial effects on the composition of the fecal microbiota (Sandri et al., 2017; Schauf et al., 2018; Bresciani et al., 2018). The diet used in our study is considered a high-protein, high-fiber diet (Table 1). Although we cannot exclude that changes observed in the fecal microbiota of dogs with obesity after weight loss are associated to the new diet, minor changes have been reported when an increase of fiber is included in the diet of healthy dogs (Middelbos et al., 2010).

It has been hypothesized that increased satiety, an important factor to aid weight loss, could be mediated by short-chain fatty acids (SCFAs) (Arora, Sharma & Frost, 2011). Our results confirm significant differences in SCFAs-producing bacteria, as Clostridiaceae, Veillonellaceae and *Blautia* between obese before and after weight loss (Data S2). However, study of SCFAs in obese dogs and after weight loss it is necessary to confirm its role in satiety and hence, in weight modulation.

## Conclusions

In summary, this study shows that the fecal microbiota of dogs with obesity significantly changes after weight loss. In addition, our results by qPCR show that after weight loss with a high-fiber and high-protein diet, the abundance of the bacterial population analyzed are mostly within the reference intervals for clinically healthy dogs.

##  Supplemental Information

10.7717/peerj.9706/supp-1 Supplemental Information 1 Dogs enrolled in the study

10.7717/peerj.9706/supp-2 Supplemental Information 2 Median percentage of relative abundance of the different bacterial populations detected in fecal samples of dogs with obesity before and after weight loss

## Additional Information and Declarations

### Data Availability

The following information was supplied regarding data availability: Raw sequences are available in NCBI GenBank: PRJNA580258.
